# Searching target sites on DNA by proteins: Role of DNA dynamics under confinement

**DOI:** 10.1093/nar/gkv931

**Published:** 2015-09-22

**Authors:** Anupam Mondal, Arnab Bhattacherjee

**Affiliations:** Center for Computational Biology, Indraprastha Institute of Information Technology (IIIT) Delhi, New Delhi-110020, India

## Abstract

DNA-binding proteins (DBPs) rapidly search and specifically bind to their target sites on genomic DNA in order to trigger many cellular regulatory processes. It has been suggested that the facilitation of search dynamics is achieved by combining 3D diffusion with one-dimensional sliding and hopping dynamics of interacting proteins. Although, recent studies have advanced the knowledge of molecular determinants that affect one-dimensional search efficiency, the role of DNA molecule is poorly understood. In this study, by using coarse-grained simulations, we propose that dynamics of DNA molecule and its degree of confinement due to cellular crowding concertedly regulate its groove geometry and modulate the inter-communication with DBPs. Under weak confinement, DNA dynamics promotes many short, rotation-decoupled sliding events interspersed by hopping dynamics. While this results in faster 1D diffusion, associated probability of missing targets by jumping over them increases. In contrast, strong confinement favours rotation-coupled sliding to locate targets but lacks structural flexibility to achieve desired specificity. By testing under physiological crowding, our study provides a plausible mechanism on how DNA molecule may help in maintaining an optimal balance between fast hopping and rotation-coupled sliding dynamics, to locate target sites rapidly and form specific complexes precisely.

## INTRODUCTION

Many *in vivo* enzymatic processes such as transcription, repression, activation etc. are triggered by specific binding of proteins to their respective target sites on DNA. A huge effort has previously been employed (1,2) to understand how these DNA binding proteins (DBPs) find their target sites rapidly, given the enormous background of non-specific DNA sequences (3–5). A plausible mechanism of ‘facilitated diffusion’ (6,7) was proposed in which DBPs were assumed to speed up their target search process by lowering their search dimension (8,9). Initially, they bind non-specifically to a random DNA site and then perform one-dimensional (1D) diffusion such as sliding, hopping along the DNA contour to reach target sites rapidly.

The details of this multifaceted search mechanism were thoroughly investigated by both experimental and *in silico* simulation techniques at various levels of complexity (10–15). For example, Clore *et al*. has performed a series of NMR experiments (16–19) to explore the fascinating spiral sliding motion and other search mechanisms. Similar protein dynamics was captured also through coarse-grained computer simulations (20). The major advantage of these techniques is to lower computation cost without implementing complex algorithms (21,22) yet probe in longer time scale to predict how search dynamics is altered by factors like conformational flexibility, sequence composition of DBPs, presence of disordered tails and the crowding agents associated with genomic DNA that might obstruct free 1D diffusion along DNA contour (23–29). It is interesting to note that these issues are mostly protein centric and only few have aimed to probe the role of DNA conformation on protein DNA interactions (30–33). One such example is that, through all-atom simulation, DNA molecule was captured switching its conformation while binding with sex determining region Y (SRY) protein (34). The role of DNA topology (35) and geometry is further underscored from the fact that even nuances in DNA conformation can remarkably change its affinity to proteins (36–42). Along the line, our previous studies have emphasized the impacts of DNA groove geometry and its determining factors on the search dynamics of DBPs (43,44). We found that, two parameters, namely the helical twist and curvature can alter DNA major groove widths and consequently the associated electrostatic potential. The interacting DBPs sense these modulations to select search mode accordingly.

In present study, we go further and deal with inherent DNA dynamics, which is also known to produce deformation in DNA groove geometry and structure (45–48). DNA dynamics however, can be highly constrained inside living cell due to macromolecular crowding (49–51). Even self-crowding of DNA due to its high packing density alters its shape and biological functions (52). Crowding is also found to facilitate diffusion of interacting proteins along the DNA contour and influence DNA replication by enhancing the activity of DNA helicase while lowering the sensitivity of DNA polymerase to salt condition (53–58). Notwithstanding this wealth of information, the interplay between molecular crowding and DNA dynamics are yet to explore, in particular, how they, in combination regulate the target search dynamics of DBPs. We enquire these issues by performing coarse-grained molecular dynamic simulations, where the DNA dynamics is modulated systematically through a simple caging potential. This ensures movement of DNA within a cylindrical cage of defined radius that mimics the available intracellular space (large R corresponds to greater intracellular space and weaker confinement/crowding). By analysing our results, we propose that DNA dynamics, together with its degree of confinement, modulates groove geometry and protein–DNA inter-communications, which in turn regulates the target search mechanism and efficiency of DBPs. Furthermore, we design kinetic experiments to confirm the crucial role played by crowded cellular environment in finding target sites and forming specific protein–DNA complexes that are key to many cellular enzymatic reactions.

## MATERIALS AND METHODS

### Protein model

The protein molecule was represented by one bead per amino acid, placed at respective C_α_ position (see Supplementary Figure S1). The conformational energy was estimated by using a native topology based model (59,60) in which a Lennard–Jones potential favoured the formation of contacts found in folded structure of the protein. In addition, electrostatic interaction was considered among negatively charged Asp and Glu and positively charged Arg and Lys residues and was modelled by Debye–Hückel potential (61,62). Despite several limitations such as lack of ion condensation effect and applicability only at low ion concentrations (63), Debye–Hückel potential has been used successfully to investigate RNA folding (64–66), chromatin assembly (67) and protein–DNA binding (68,69). To this end, we noted that the level of coarse graining also influences electrostatic interactions. The effective electrostatic in our model was weaker because of longer inter-bead distances compared to atomistic models and therefore lower salt conditions were required to allow strong interactions between protein and DNA molecules (26,70) compared to typically used salt concentrations in experiments.

### DNA model

For DNA, we adopted 3SPN.1 model developed by Pablo *et al*. (71), in which three beads were used to represent phosphate, sugar and nitrogenous base of each nucleotide and were positioned at their respective centers. Unit negative charge was placed over phosphate beads and related electrostatic interaction was modelled through Debye–Hückel potential. Further details of the model can be found in Supplementary text and in original paper. The model has been successful in estimating dsDNA persistence length with respect to ionic strength, describing melting temperature as a function of composition and ionic strength. Importantly, the same model was recently adopted by Terakawa *et al*. (72) to investigate p53-DNA non-specific interactions.

The DNA confinement was modeled by assuming a spherical cylinder (73,74) around DNA molecule along z-direction. An associated caging potential *V_cage_* ensured that DNA molecule would be restricted inside the cylinder of radius R. The form of caging potential is given by (73),

\begin{equation*} \begin{array}{*{20}l} {V_{cage} = } \\ {\sum\limits_i {K_{cage} \left[ {\left( {\frac{C}{{2(R - d_i )}}} \right)^4 - 2\left( {\frac{C}{{2(R - d_i )}}} \right)^2 - 1} \right]} H\left( {\frac{C}{2} - (R - d_i )} \right)} \\ \end{array} \end{equation*}

where, R is radius of cylinder, *d_i_* is distance between center of any DNA base pair from linear DNA axis that passes through two constrained ends of DNA. *H (x*) is Heaviside function, given by *H (x)* = 1 for *x* > 0 and *H (x)* = 0 otherwise. *K_cage_* is set as 100.0 and C = 4.0 Å. A clear advantage of this kind of confinement over the use of many spherical inert crowders is to lower computation cost significantly yet effectively mimic crowded cellular environment.

### Simulation protocol

The initial B-DNA structure of a random 200 base pair (bp) sequence was generated in w3DNA (3D DNA structure) web server (75). The folded structure of 93-residue long Sap-1 was obtained from (PDB Id: 1BC8) Protein Data Bank that features a helical recognition region (53 to 68 residues, see red region in Supplementary Figure S1) to scan DNA molecule. We started by placing the molecules inside a simulation box of size 220 Å × 220 Å × 820 Å with periodic boundary condition and DNA being oriented along Z-axis. The time evolution was studied through Langevin dynamics with friction coefficient γ = 0.25 and temperature T = 300 K. The protein molecule was allowed to interact non-specifically with DNA through excluded volume and electrostatic interactions. Salt condition (*Cs*) was varied from 20–200 mM to investigate salt dependence of target search dynamics. Production runs were typically 10^8^ MD steps long during which multiple associations between protein and DNA molecules were observed. The relative interaction strengths were such that the Sap-1 remained completely folded at simulation temperature, while the DNA model was kept unchanged to maintain its physical properties. It should be noted that during our simulations only DNA was under confinement and not the protein. This helped to investigate the interplay of degree of confinement with DNA dynamics in isolation. Crowding effect on protein is however, only to force it to be in close proximity of DNA molecule, which can also be achieved by lowering salt condition and thereby increasing the effective electrostatic attractions between protein and DNA.

During kinetic experiments we monitored formation of specific protein–DNA complex. For that we inserted the DNA target sequence found in crystal structure of Sap-1, at the center of 200 bp DNA sequence. The specific contacts between protein (at the C_α_ level) and DNA molecules were then identified and modeled through a soft attractive Lennard–Jones potential. In addition, Sap-1 was allowed to interact non-specifically with DNA bases outside the target region. We modelled this by another short-ranged Lennard–Jones potential, where the bases could randomly interact with protein residues, belonging to its recognition region. Associated interaction strength (ϵ_ij_) was chosen from a Gaussian distribution (F (ϵ) = (1 / (2πσ^2^)^1/2^) exp [− ((ϵ − ϵ_nonspecific_)^2^ / 2σ^2^)]) with mean (ϵ_ij_) = 0.6 and standard deviation (σ) of 0.1 (76). To achieve statistical robustness for protein–DNA systems with different degrees of DNA confinement, we performed 300 independent simulations, each 5 × 10^6^ MD steps long.

### Sliding, hopping, 3D diffusion and 1D diffusion coefficient

The structural discrimination between various search mechanisms, namely sliding, hopping and 3D diffusion were done by following the methods prescribed in previous works (20,43,44) and is discussed briefly here. We assumed that protein molecule was performing 3D diffusion if center of its recognition helix was more than 30 Å away from center of closest DNA base pair. A snapshot was defined as sliding mode if at least 70% of recognition region was in contact with DNA major groove, center of mass of recognition region was with in 18 Å from the center of closest DNA base pair and orientation angle (Ψ) was <25°. This ensured closest approach and proper orientation of proteins to the DNA sites, which is essential to form specific protein–DNA complexes. If recognition helix was found at a distance of less than 30 Å from DNA and yet did not match any of the sliding criteria, we considered the protein performing hopping along DNA. The 1D diffusion coefficient D1 was measured from linear behaviour of mean square displacement of Sap-1 along DNA contour while performing sliding and hopping only.

## RESULTS AND DISCUSSIONS

**Figure 1.**
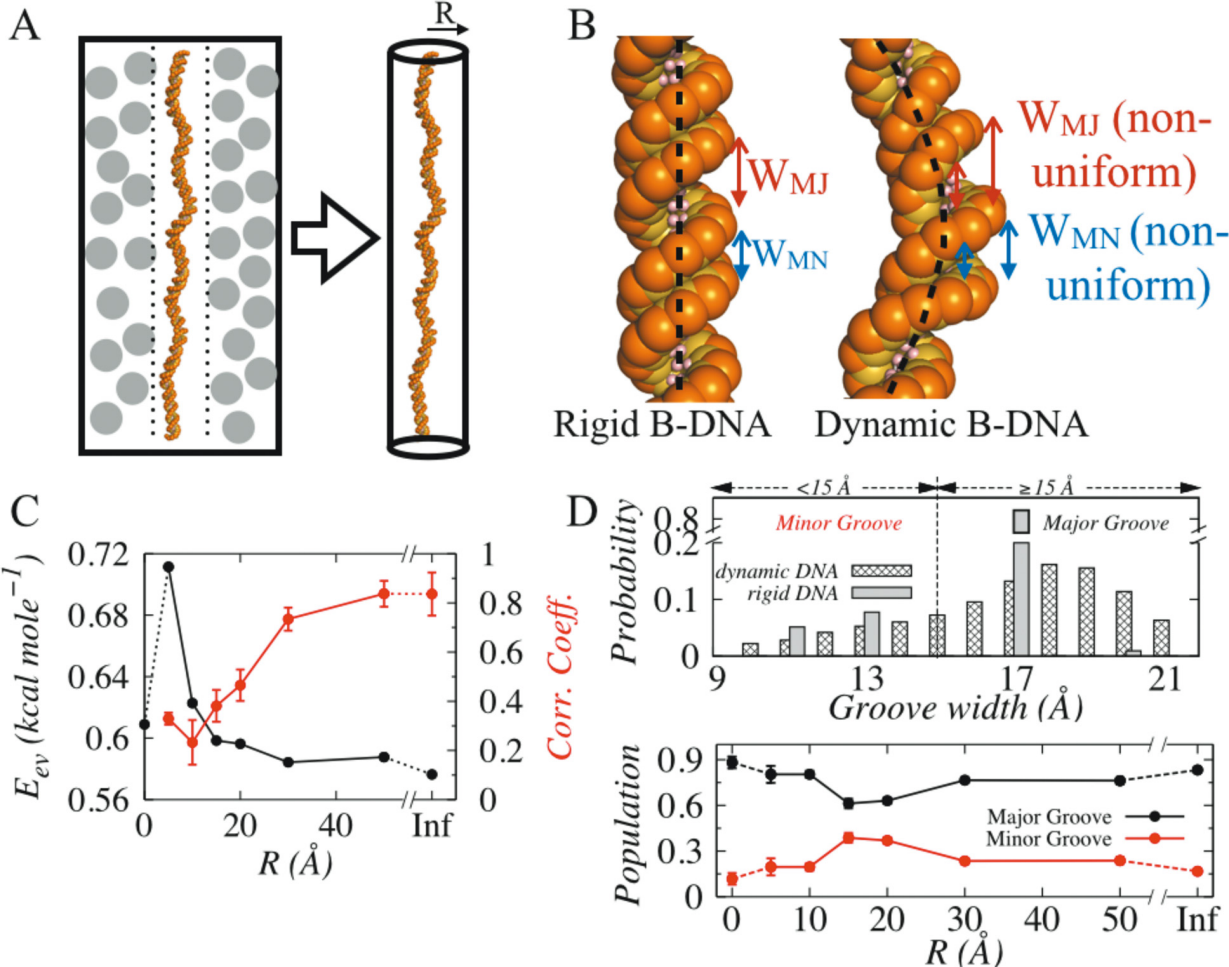
Structural characterization of rigid and dynamic DNA molecules. (**A**) The impact of cellular crowding on DNA can be effectively captured by a spherical cylinder of radius R associated with a caging potential *V_cage_*. (**B**) Dynamics induced structural deformation in rigid and dynamic B-DNA molecules (partly shown). Unlike B-DNA geometry, major (*W_MJ_*) and minor (*W_MN_*) groove widths vary significantly in dynamic DNA coupled with commonly observed bent DNA axis. (**C**) Variation of steric clashes (*E_ev_*, denoted black line) and correlation in dynamics of interacting molecules (red line) as function of intracellular space R at 20 mM. Depending on the degree of DNA confinement (indicated by R), these two parameters jointly determine the inter-communication between protein and DNA molecules. (**D**) Correlations between the occupancy probability of major (black) and minor (red) grooves with the degree of DNA confinement.

In order to investigate how DNA dynamics under various degree of confinement modulates target search dynamics and kinetics of DBPs, we performed coarse-grained molecular dynamics simulations of Sap-1 with a randomly selected 200 bp DNA molecule that was encapsulated in a cylinder (Figure 1A). The radius (R) of the cylindrical cage that mimics available intracellular space for DNA molecule was varied between 5–50 Å. In addition, a zero value of R indicates rigid conformation with ideal B-DNA geometry and R-value assigned as infinity (Inf) signifies absence of caging potential and DNA molecule was fully flexible without any space restriction on its dynamics. We first identified molecular determinants that govern the protein–DNA inter-communication for a fixed salt condition but different degrees of DNA confinement, followed by a thorough investigation on how these key components regulate the intricate details of various search mechanisms and search efficiency of DBPs. All the results were compared with interactions of Sap-1 and rigid B-DNA to underscore the influence of DNA dynamics.

### DNA dynamics and its degree of confinement regulate groove geometry and inter-communication with proteins

Clearly, dynamic DNA has more degrees of freedom compared to rigid B-DNA. The structural fluctuation caused by its inherent dynamics often results into bent DNA axis and non-uniform groove geometry (Figure 1B) unlike to rigid B-DNA molecule that features linear DNA axis and constant major and minor groove widths. For example, average widths of major and minor grooves in dynamic DNA molecule without any confinement are 17.97 ± 0.72 Å and 13.63 ± 1.68 Å, respectively (Supplementary Figure S2) compared to 17.25 Å and 11.58 Å of rigid B-DNA. Similar kind of deformation, particularly in minor groove has been reported previously and was found to be essential for interaction with other biomolecules and intercalation of drugs (48,77). Another factor that governs inter-communication between protein and DNA molecule is the strength of non-specific electrostatic interaction, which is however, roughly the same for cases with fixed salt concentration. Under this condition, we found two factors, namely (i) steric clashes (related energy *E_ev_*) and (ii) degree of correlation between dynamics of protein and DNA that determine their inter-communications. The *E_ev_* term excludes repulsive interaction between DNA molecule and wall of the cage. The degree of correlation between dynamics of protein and DNA was measured by monitoring positions of center of recognition region of protein and closest DNA bp from it with time. Our result suggests (Figure 1C) that with increase in R, correlation between dynamics of protein and DNA increases, whereas *E_ev_* decreases. This can be rationalized from the fact that at low values of R, when DNA is tightly confined and only limited space is available to move, it frequently hits the wall of cage and reverts back. While doing so, it also encounters and collides with protein that approaches towards it through strong electrostatic interactions. This leads to high *E_ev_* (higher number of steric clashes) but low correlation in their dynamics as their movements are in opposite direction. With increase in R, available space for DNA movement increases and protein follows it via non-specific electrostatic interaction, resulting decrease in number of steric collisions (low *E_ev_* values) but rise in correlation between their dynamics (up to 80%). The initial increment in *E_ev_* is due to intra-DNA excluded volume interaction in dynamic DNA, which is constant in rigid B-DNA.

Having recognized the determinants that regulate communications between protein and DNA molecules (*E_ev_* and correlation in dynamics), we intended to examine how they together with the deformations in DNA groove geometry affect target search dynamics of DBPs. One recent study has suggested that steric collisions may facilitate 1D diffusion of DBPs (78). The model however, was oversimplified (protein was presented by three beads and pair of nucleotide by one bead only) to capture important details of search mechanisms. To this end, resolution of our model is found to be adequate to monitor how Sap-1 scans DNA contour non-specifically. Typically, it spends major fraction of its time inside the wider major grooves of rigid B-DNA and occasionally hops at narrower minor grooves. Situation changed when DNA dynamics was taken into account. In the upper panel of Figure 1D, we presented distribution of groove widths occupied by Sap-1 during our simulations with rigid and dynamic DNA without confinement, which suggests that minor groove (<15 Å) occupancy for Sap-1 in dynamic DNA is 4% higher (∼16%) compared to that in rigid DNA (∼12%). Greater changes can be observed with inclusion of DNA confinement. The lower panel of Figure 1D presents major and minor groove populations as functions of intracellular space, R. With decrease in degree of DNA confinement (from R = 5–20 Å), minor groove population rises by ∼18% (from 19% to 37%). Thanks to the rapid drop in steric collisions (*E_ev_* reduces by ∼0.12 kcal/mole) and poor correlation (<40%, see Figure 1C) between dynamics of interacting molecules that often lands Sap-1 adjacent to minor grooves, and as they are wider in dynamic DNA compared to rigid B-DNA, Sap-1 fits more comfortably with lower steric hindrance. For R > 20 Å, when DNA confinement effect is weak, strong electrostatic interactions (at 20 mM) and high correlation between dynamics of protein and DNA guides the former smoothly towards wider major grooves, resulting in increment of major groove populations. One should note here, that mere scanning at major and minor grooves do not lead to formation of bound state unless sliding criteria are satisfied (see Materials and Methods).

### Protein dynamics on rigid and dynamic DNA as a function of salt concentration

**Figure 2.**
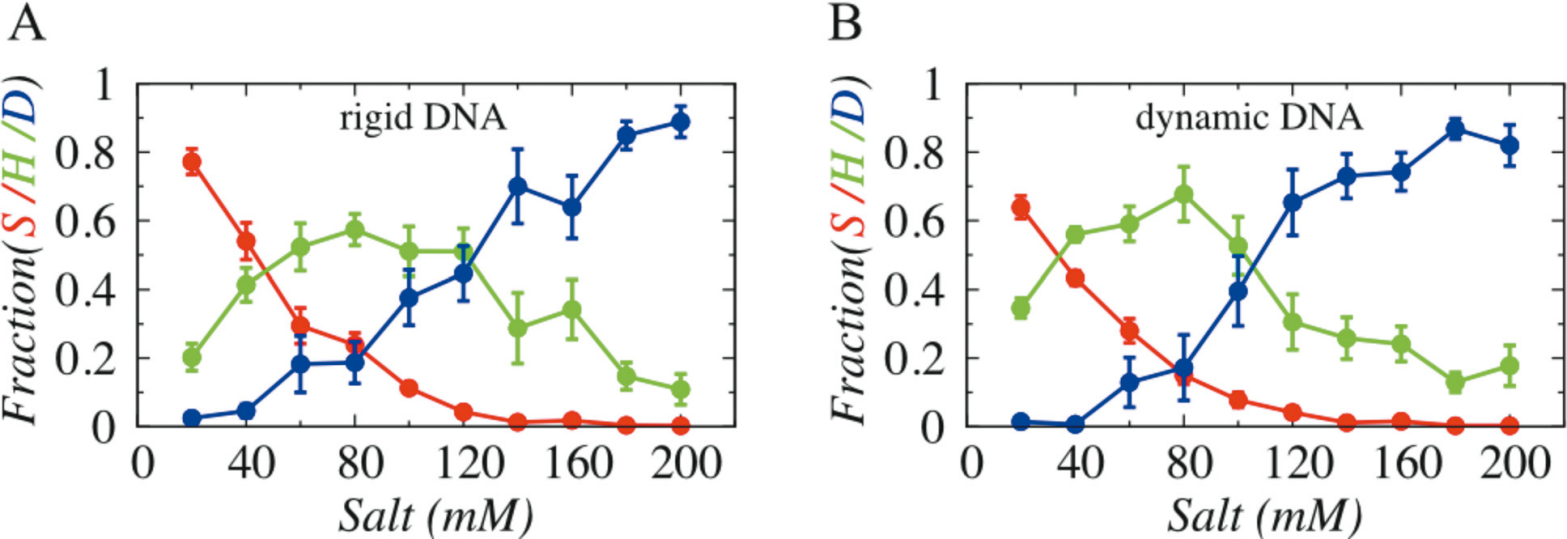
Effect of salt concentration on the interplay between sliding (S), hopping (H) and 3D diffusion (D) adopted by Sap-1 on (**A**) rigid DNA molecule and (**B**) dynamic DNA molecule.

Before proceeding further to investigate how the changes in major and minor groove populations were translated into altered search mechanisms in DBPs, it was instructive to identify suitable experimental conditions at which complete diversity of various search mechanisms could be explored. Previous studies have indicated that salt condition plays a major role in screening Debye–Huckel electrostatic interactions and thereby modulates the propensities of different search mechanisms such as sliding, hopping and 3D diffusion. Therefore, to recognize representative salt conditions, we performed two sets of coarse-grained molecular dynamics simulations of Sap-1 with 200 bp long rigid and dynamic DNA without confinement, under a wide range of salt concentrations (20–200 mM). By analysing the structural details of each snapshot evolved during simulations, we estimated propensities of various search techniques as functions of salt conditions (Figure 2).

Our result suggests that overall profiles of various search techniques are very similar for both rigid and dynamic DNA without confinement. The sliding propensity decreases while 3D diffusion increases with increase in salt concentrations. This result is in agreement with previous observations (20,43) and can be explained as high salt concentrations weaken the non-specific electrostatic attractions between protein and DNA, resulting easier dissociation of proteins (increase in 3D diffusion) from DNA surface. The hopping mode, during which proteins bind not so strongly with DNA as they do in sliding dynamics, is most populated at moderate salt conditions (∼80–100 mM). Further inspection of the plots showed that at very low salt condition (20 mM), Sap-1 predominantly performs 1D diffusion (∼77% sliding and ∼20% hopping on rigid DNA, whereas ∼64% sliding and ∼34% hopping on dynamic DNA) and only few 3D diffusion (less than 3%) events. In contrast, at 100 mM, sliding propensity of Sap-1 diminishes and 3D diffusion increases significantly (∼38% for rigid DNA and ∼39% for flexible DNA), making the 1D–3D search mechanisms comparable irrespective of rigid and dynamic DNA. We, therefore, selected these two salt conditions, which offer complete diversity of various search mechanisms of proteins.

### Protein dynamics changes with degree of DNA confinement

**Figure 3.**
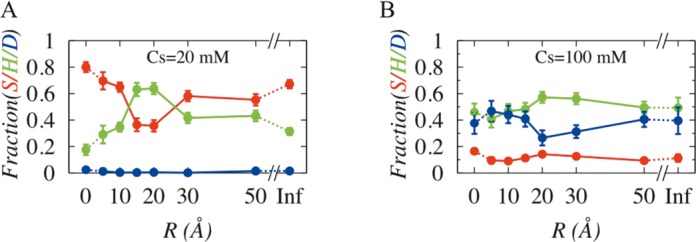
Effect of degree of DNA confinement on search dynamics (sliding (S), hopping (H) and 3D diffusion (D)) of DBPs at (**A**) 20mM and (**B**) 100 mM.

Having identified suitable salt conditions (20 mM and 100 mM), we turned to probe the influence of DNA confinement on dynamics of DBPs by performing extensive simulations of Sap-1 with DNA molecule being placed inside cylindrical cage of various radii. The sampled conformations of protein–DNA pairs during simulations were examined to measure propensities of sliding, hopping and 3D diffusion with respect to R and are presented in Figure 3A and B at 20 mM and 100 mM, respectively. We found that at 20 mM, sliding propensity decreases and hopping increases initially (by ∼50% compared to rigid DNA) and even becomes preferred mode (associated probability is higher compared to sliding dynamics) of transport at R = 15–20 Å. Beyond R = 20 Å, reverse trend is observed. This profile is very similar to the major and minor groove occupancy profiles given in lower panel of Figure 1D. Under strong to moderate confinement regime (R ≤ 20 Å), rise in minor groove population promotes hopping dynamics, while increment in major groove populations beyond R = 20 Å directly corresponds to rise in sliding propensity. At 100 mM, weakening of electrostatics results sharp fall in sliding probability, and as an alternative 3D diffusion mode competes with hopping dynamics. The initial gain in hopping propensity under strong to moderate DNA confinement (R ≤ 20 Å) is due to higher number of steric clashes (high *E_ev_*, see Supplementary Figure S3 and S4) that forces DNA to frequently encounter Sap-1 even though the correlation between their dynamics is extremely poor. For R > 20Å, number of steric clashes decreases, which along with poor correlation in dynamics between protein and DNA ensures no inter-communication between these molecules and consequently 3D diffusion propensity rises.

### Degree of DNA confinement determines mechanistic details of sliding dynamics

The degree of DNA confinement also plays crucial role in altering mechanistic details of different search mechanisms. To analyse such effects, we investigated structural details of all snapshots in which Sap-1 slides along DNA contour and monitored how rotational angle (θ) and transversal displacement (along Z-axis) of the protein vary.

**Figure 4.**
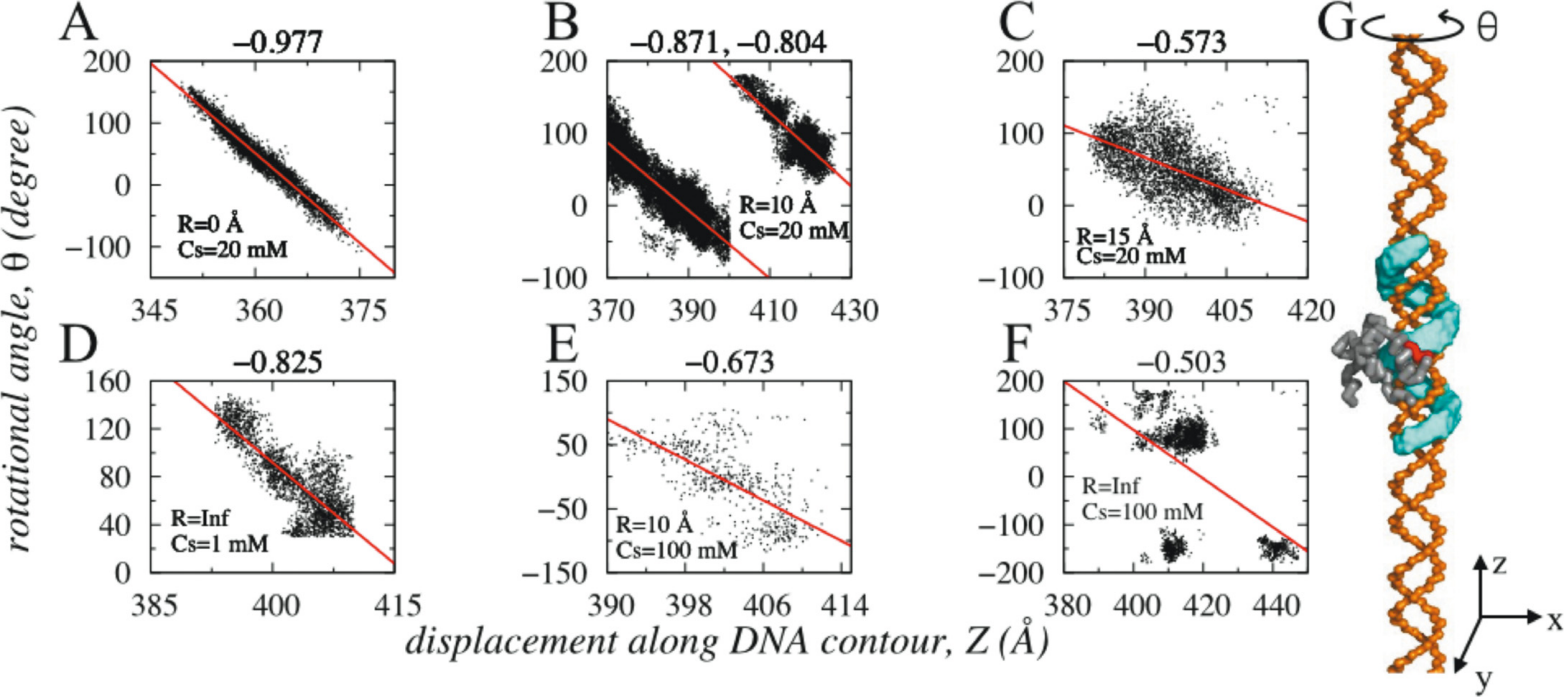
Correlation between transversal (Z-axis) and rotational (θ) motions of Sap-1 at various salt conditions and degree of DNA confinement. For fixed salt condition (20 mM), correlation coefficient (mentioned on the top of each figure) decreases with increase in R (**A**–**C**). (**B**) indicates fragmented sliding events (discussed further in Figure 5) at two distinctly different major grooves separated by ∼34 Å. The correlation however, can be high (**D**) even on dynamic DNA without confinement if the salt condition is extremely low (1 mM). At higher salt condition (100 mM) reasonable correlation is found for strong confinement (R = 10 Å in **E**), which reduces significantly if the confinement effect is not imposed (**F**). The red lines in each figure represent the best fits. Figure 4G presents the sample trace (cyan color) of rotation coupled sliding performed by Sap-1 along the major grooves of rigid DNA molecule.

In Figure 4, we presented the variation of θ with displacement of Sap-1 along Z-axis under different degrees of confinement and salt conditions and estimated correlation between them. A high magnitude of correlation coefficient indicates that displacement along DNA contour (Z-axis) is strongly affected by the helicity of DNA, which means that while diffusing along DNA, Sap-1 simultaneously rotates by inserting its positively charged recognition region inside the negatively charged phosphate atoms surrounded major grooves (see Figure 4G). Our results suggest that such rotation-coupled sliding can be achieved either if (i) DNA is rigid/under strong confinement or (ii) if salt concentration is low. For example, large correlation between rotational and translational motion of Sap-1 can be seen for R < 15 Å, even at high salt condition, such as for R = 10 Å and 100 mM (Figure 4E). This is because under strong confinement, both DNA helicity and groove geometry are minimally perturbed, which helps protein to perform rotation-coupled sliding. Conversely, under weak confinement, DNA dynamics can result in significant bending of DNA axis and associated non-uniformity in DNA helicity and groove widths prevent rotation-coupled sliding. In fact, our previous study indicated that protein performs rotation-decoupled sliding if DNA has bent topology (circular DNA) (44). However, at extremely low salt concentration, (1 mM in Figure 4D) Sap-1 performs rotation-coupled sliding even though confinement effect was not imposed. The associated electrostatics at this salt condition is extremely strong to force Sap-1 to remain tightly bound with DNA and slide by ignoring the fluctuations in DNA conformation. This is supported by a recent computational study under same salt condition (72).

### DNA confinement promotes many but short sliding events

**Figure 5.**
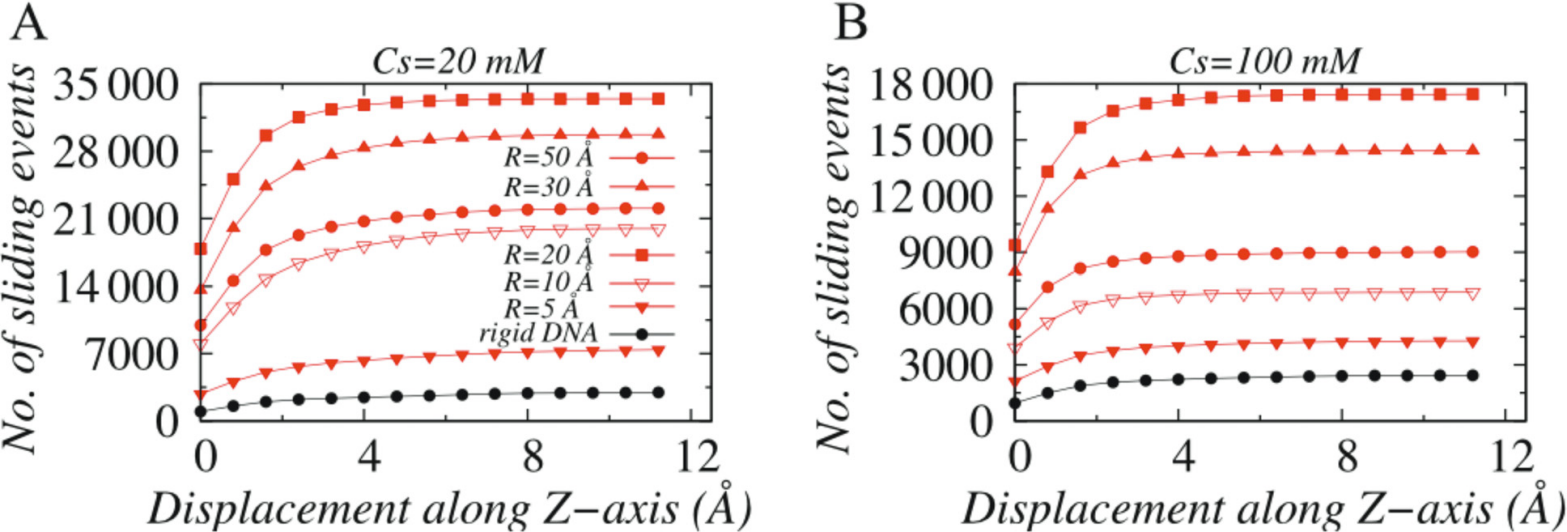
Variations in number of cumulative sliding events as a function of displacement along Z-axis at salt concentration (**A**) 20 mM and (**B**) 100 mM.

Furthermore, the number and length of sliding events also vary with degree of DNA confinement. By sliding event we mean a time stretch during which Sap-1 continuously probed DNA through sliding only. Between two sliding events, protein can perform hopping and 3D diffusion. The length of each sliding event is measured from Z-axis displacement during an event. In Figure 5A and B, we estimated cumulative number of sliding events performed by Sap-1 on DNA under various degree of confinement at 20 mM and 100 mM, respectively. Our results suggest that with increase in R, hopping (Figure 3) and rotation-decoupled sliding propensity favour fragmented (more in number) sliding events (∼33 000 sliding events near R = 20–30 Å compared to only ∼2900 on rigid DNA). The frequent disruption leads to shorter sliding length that measures how far protein moves during a single sliding event. For example, Sap-1 moves ∼10–11 Å along Z-axis through rotation coupled sliding dynamics on rigid DNA whereas, the same on dynamic DNA with weak confinement (R = 20 Å) is merely ∼7–8 Å. This indicates that decreasing DNA confinement promotes many but short rotation-decoupled sliding events. For R > 20–30 Å, sliding events are less disruptive due to fall in hopping propensity that corresponds fewer but comparatively longer sliding events. Similar trend can be observed at 100 mM except the fact that overall sliding propensity is much weaker (see Figure 3B) and is reflected in number of sliding events, which is only ∼0.50% (∼17 000) to that in 20 mM.

### Efficiency of DNA search vary with degree of DNA confinement

How the changes in mechanistic details of sliding dynamics with variation in degree of DNA confinement influence target search efficiency of DBPs? Previously, Givaty and Levy have investigated search efficiency of proteins, defined by the numbers of DNA base pairs scanned during sliding motion, as a function of salt condition. Using a coarse grained rigid DNA model, they concluded that while sliding is must-have in order to form specific protein–DNA complexes; this mode of translocation is typically slow. In fact, experiment has also indicated that on average DBPs take more than 200 ns to slide a single base pair (12). In comparison, hopping dynamics is faster and a right blend of sliding and hopping may result rapid 1D diffusion with high precision in forming specific complexes. This is further supported by a separate study by Berg *et al*. (6).

**Figure 6.**
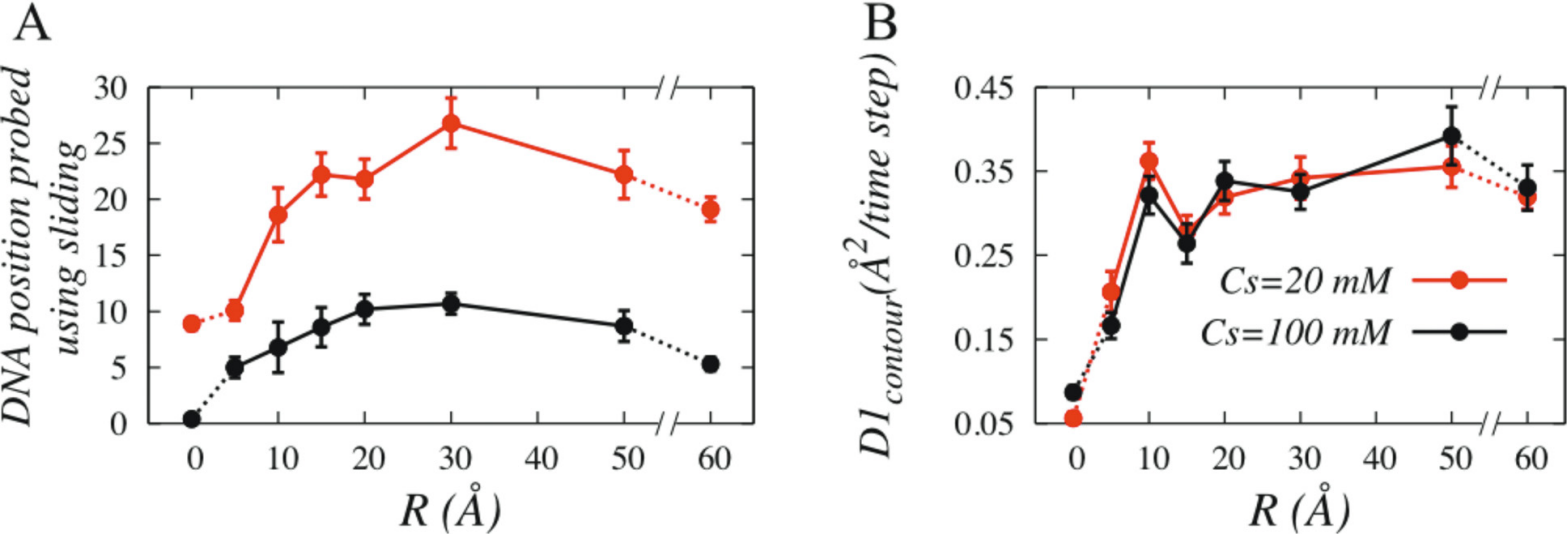
Role of DNA confinement on (**A**) the target search efficiency of DBPs, defined as the number of base pairs traversed using sliding dynamics at salt concentration of 20 mM (red) and 100 mM (black), and (**B**) on 1D diffusion coefficient. Diffusion coefficient was estimated from the linear behaviour of the mean square displacement of Sap-1 through sliding and hopping dynamics.

In order to estimate search efficiency of Sap-1, we measured the number of DNA sites probed by Sap-1 during sliding motion at 20 mM and 100 mM and is presented in Figure 6A. With increase in R, hopping propensity increases that effectively speed up the 1D diffusion on Sap-1 while during fragmented sliding events it reads DNA sites. The faster diffusion is also reflected from initial rise in 1D diffusion coefficient in Figure 6B. At R = 30 Å Sap-1 reads maximum number (∼27) of DNA base pairs under 20 mM, which is approximately three times more than it could scan when the DNA is rigid. Further weakening of DNA confinement leads lowering in hopping dynamics that reduces 1D diffusion of Sap-1, which then could only visit to fewer DNA sites. Similar behaviour is observed at 100 mM, however, the maximum number of positions probed is roughly 1/3 compared to that at 20 mM because of sharp decrease in overall sliding propensity.

### Role of DNA confinement in facilitated diffusion of proteins

It is interesting to note that the number of probed position on dynamic DNA without any confinement is ∼2–5 times higher compared to rigid DNA. Even the maximum search efficiency is obtained at R = 30 Å, which corresponds to relatively weak DNA confinement. Does it mean that DBPs could exhibit more efficiency had the cellular environment been least crowded? To test this, we performed kinetic experiments at 20 mM where Sap-1 was initially placed 20 bp away from the target site and 30 Å away from the DNA surface to avoid biased search by electrostatic interaction. We considered three systems; rigid B-DNA, dynamic DNA with no confinement and DNA with confinement (R = 15 Å) and monitored how the different degrees of DNA confinement affect the time to locate DNA target sites by Sap-1 and thereby forming the specific protein–DNA complex. The progress of association was monitored by <*Q_sp_*> that denotes the fraction of specific contacts, averaged over all simulations that lead to formation of specific protein–DNA complex.

**Figure 7.**
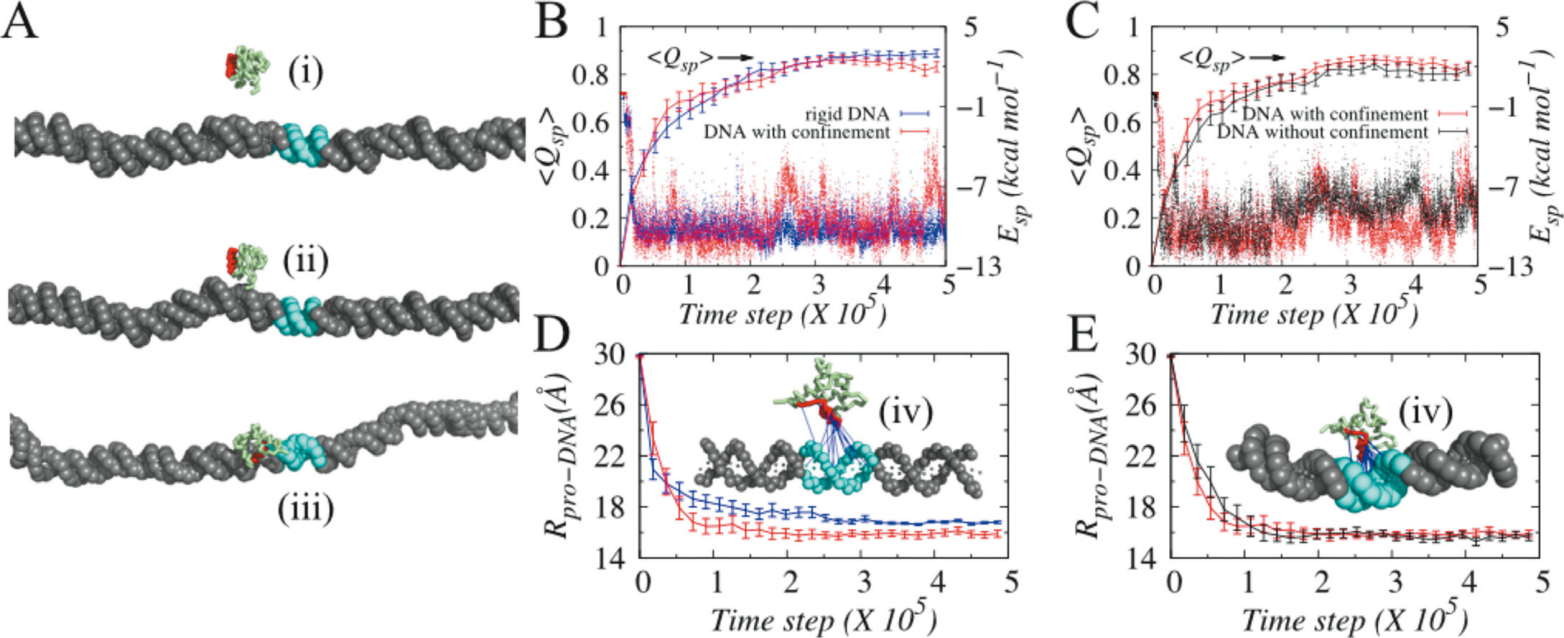
Kinetics of specific protein–DNA complex formation. (**A**) Representative snapshots explaining step-by-step transition from (i) unbound to (ii) non-specifically bound to (iii) specifically bound state. (**B**) and (**C**) present the fraction of average specific contacts (<*Q_sp_*>, solid lines) and sample specific energies (E_sp_) as functions of time for rigid DNA (blue line), dynamic DNA under strong confinement (R = 15 Å, red line) and dynamic DNA without confinement (black line). (**D**) and (**E**) present the corresponding evolution of distances (R_pro-DNA_) between center of recognition region of Sap-1 and center of closest DNA base pair. The final DNA bound conformations of Sap-1 with rigid B-DNA and dynamic DNA with confinement are shown in the insets of (**D**) and (**E**).

Our result suggests that in absence of non-specific contacts, Sap-1 is extremely quick in finding the target site on rigid DNA (up to ∼2.6 times faster, Supplementary Figure S5) compared to when DNA is dynamic. This is because of smooth rotation coupled sliding ability of Sap-1 on rigid DNA that helps it to probe DNA sites continuously and recognize target site precisely. However, it is argued that during one-dimensional search along DNA, sequence-specific proteins may come in close contacts and therefore may interact non-specifically with DNA sites that shares at least partial sequence similarity with the target one. Typically, such non-specific interaction slows down sliding dynamics (76). In deed, by using random walk models, Slutsky *et al*. have shown that heterogeneous energy landscape is common to biological systems such as protein–DNA, where variability to the energy of interactions correlated over the protein footprint size greatly diminishes efficiency of sliding (79). When considered, we also found that nonspecific interaction delays target search kinetics (by ∼41%, MD steps required corresponding to <*Q_sp_*> = 0.5 in Supplementary Figure S5 is given by ∼0.35 × 10^5^) in rigid B-DNA (blue line in Figure 7B) and is comparable to that with dynamic DNA under confinement (red line). For both systems, ∼0.6 × 10^5^ MD steps were needed to achieve <*Q_sp_*> = 0.5. The effect of non-specific interaction is weak in dynamic DNA where, hopping propensity is considerably high. The ability to hop frequently not only allows Sap-1 to get rid of being trapped at local energy minima associated with non-specific contacts, it also let sap-1 to quickly orient at the target site to form specific protein–DNA complex. This is evident from the fact that 65% of all specific contacts were formed in dynamic DNA with confinement by ∼0.7 × 10^5^ MD steps (Figure 7B), which is 1.5 times faster compared to rigid B-DNA. However, the hopping propensity is related to degrees of DNA confinement and under weak or no confinement (black line, Figure 7C), too much of hopping often results Sap-1 in jumping over the target site to miss it and sometimes coming back later. This caused target search delayed and ∼17–19% less probable in dynamic DNA without confinement compared to that in rigid B-DNA. Once the specific complex is formed (<*Q_sp_*> > 0.8), a dynamic bond formation and breaking process can be realized from fluctuating <*Q_sp_*> values in dynamic DNA with confinement. This stems from the interplay between structural fluctuations in dynamic DNA and affinity to form specific protein–DNA complexes. With rise in binding affinity (Supplementary Figure S5), such behaviour fades out. One interesting trait observed in our kinetic experiment is that even though the target search kinetics on rigid B-DNA and on DNA with confinement are comparable, the former lacks conformational flexibility to achieve desired specificity. For example, in absence of structural flexibility in DNA, Sap-1 could not approach the target DNA site precisely and was forced to remain at a larger distance from DNA (high R_pro-DNA_ for rigid B-DNA in Figure 7D and corresponding bound structure is given in the inset of Figure 7D) and therefore formed a loosely bound complex with rigid B-DNA compared to that in dynamic DNA (tightly bound specific complex with dynamic DNA under confinement is shown in the inset of Figure 7E). Associated specific energy values are also in agreement and shows greater stability for dynamic DNA with confinement (red dots, Figure 7B). DNA confinement thus, provides a balance between speed and accuracy; at R = 15 Å, Sap-1 could perform adequate amount of rotation coupled sliding (Figure 4C) to locate target DNA site precisely and supports enough hopping dynamics to diffuse one dimensionally faster compared to dynamic DNA confinement. The DNA also possesses sufficient flexibility to orient itself rightly to form the specific protein–DNA complex, which is not achievable if the confinement is too strong such that DNA looses its structural flexibility as in rigid B-DNA.

To this end, it is desirable to translate the related degree of DNA confinement into concentration of crowders. If we assume that radius R = 15 Å means a crowder molecule to be present at least 30 Å (diameter of cylinder) away from DNA molecule and if this is the average distance between DNA and crowder molecules, the concentration of crowders would be ∼0.37 mM according to the prescription given in reference (80). In comparison, the *in vivo* concentration of cytoplasm is estimated to be 50–400 mg ml^−1^, which is ∼0.9–7 mM if the average molecular weight is assumed to be ∼55 kDa. This indicates role of physiological crowder concentration in target search dynamics of DBPs.

## CONCLUSIONS

Searching DNA target sites by DBPs is immensely complex process to understand fully. While many insights have been gained on protein part to underpin the effects of its flexibility and composition on the target search efficiency, little has been explored on DNA part. Furthermore, DNA is more commonly treated as linear rigid molecule with negatively charged surface with which protein interacts non-specifically through electrostatic interactions.

In this study, we considered DNA dynamics, which is inherent to the molecule and investigated the impact of its interplay with DNA confinement on target search dynamics of DBPs. Imposing confinement on DNA was inspired from the crowded environment of cell, which along with DNA self-crowding may impact shape and function of the molecule significantly (52). By performing extensive molecular dynamic simulations, we propose that the degree of DNA confinement and its inherent dynamics concertedly play pivotal roles in determining groove geometry of the molecule and inter-communications with protein. For example, we identified two molecular determinants namely, number of steric clashes between protein and DNA and correlation between their dynamics that govern their inter-communication. Steric clashes promote faster 1D diffusion to interacting protein by enhancing its hopping propensity. Similar observation was recently reported (78), although how the numbers of protein–DNA steric collisions are regulated by the interplay of DNA confinement and DNA dynamics was unknown. Furthermore, we observe that repulsive interaction from crowders or caging wall causes DNA to encounter nearby protein molecules frequently and thereby increases the contact time. It is noteworthy that a similar conclusion but from a different perspective mentioned that presence of crowders act as a shield to prevent the escape of interacting protein molecules and thereby increases the contact time with DNA (56,57). It may be the case that in reality, molecular crowding functions in both ways: it prevents dissociation of interacting DBPs, as well as modulates the dynamics of DNA such that it encounters nearby DBPs more often.

We further highlight the role of DNA confinement and its dynamics by investigating the details of sliding mechanism and find that decreasing degree of DNA confinement promotes many but short rotation-decoupled sliding events interspersed by hopping dynamics. While such modulation in sliding dynamics and associated high hopping propensity help DBPs to scan maximum number of DNA sites by allowing them to move faster (high 1D coefficient) over the weakly frustrated protein–DNA landscape in presence of non-specific contacts, they often lack the precision. The poor correlation between rotational and translational motion during short sliding events prevents the DBPs to scan DNA base sites thoroughly and in addition to that, high hopping propensity makes DBPs to often jump over the target DNA sites to miss it. Our simulations therefore suggest that it is not just naive speed rather mixture of speed and accuracy that causes DBPs to successfully form the specific protein–DNA complex upon recognizing the target DNA sites precisely. By providing a weighted balance between hopping and rotation-coupled sliding, DNA dynamics under physiological crowding helps DBPs to achieve this goal.

## SUPPLEMENTARY DATA

Supplementary Data are available at NAR Online.

SUPPLEMENTARY DATA

## References

[1] Mirny L., Slutsky M., Wunderlich Z., Tafvizi A., Leith J., Kosmrlj A. (2009). How a protein searches for its site on DNA: the mechanism of facilitated diffusion. J. Phys. A Math. Theor..

[2] Tafvizi A., Mirny L.A., van Oijen A.M. (2011). Dancing on DNA: kinetic aspects of search processes on DNA. Chemphyschem.

[3] Lavery R., Zakrzewska K. (2012). Towards a molecular view of transcriptional control. Curr. Opin. Struct. Biol..

[4] Garvie C.W., Wolberger C. (2001). Recognition of specific DNA sequences. Mol. Cell.

[5] Von Hippel P.H. (2007). From “simple” DNA-protein interactions to the macromolecular machines of gene expression. Annu. Rev. Biophys. Biomol. Struct..

[6] Berg O.G., Winter R.B., von Hippel P.H. (1981). Diffusion-driven mechanisms of protein translocation on nucleic acids. 1. Models and theory. Biochemistry.

[7] Von Hippel P.H., Berg O.G. (1989). Facilitated target location in biological systems. J. Biol. Chem..

[8] Riggs A., Bourgeous S., Cohn M. (1970). The lac represser-operator interaction *1, *2III. Kinetic studies. J. Mol. Biol..

[9] Halford S.E. (2009). An end to 40 years of mistakes in DNA-protein association kinetics. Biochem. Soc. Trans..

[10] Wang J., Lu Q., Lu H.P. (2006). Single-molecule dynamics reveals cooperative binding-folding in protein recognition. PLoS Comput. Biol..

[11] Gorman J., Plys A.J., Visnapuu M.-L., Alani E., Greene E.C. (2010). Visualizing one-dimensional diffusion of eukaryotic DNA repair factors along a chromatin lattice. Nat. Struct. Mol. Biol..

[12] Blainey P.C., Luo G., Kou S.C., Mangel W.F., Verdine G.L., Bagchi B., Xie X.S. (2009). Nonspecifically bound proteins spin while diffusing along DNA. Nat. Struct. Mol. Biol..

[13] Bustamante C. (1999). Facilitated Target Location on DNA by Individual Escherichia coli RNA Polymerase Molecules Observed with the Scanning Force Microscope Operating in Liquid. J. Biol. Chem..

[14] Elf J., Li G.-W., Xie X.S. (2007). Probing transcription factor dynamics at the single-molecule level in a living cell. Science.

[15] Esadze A., Iwahara J. (2014). Stopped-Flow Fluorescence Kinetic Study of Protein Sliding and Intersegment Transfer in the Target DNA Search Process.

[16] Marius Clore G. (2011). Exploring translocation of proteins on DNA by NMR. J. Biomol. NMR.

[17] Doucleff M., Clore G.M. (2008). Global jumping and domain-specific intersegment transfer between DNA cognate sites of the multidomain transcription factor Oct-1. Proc. Natl. Acad. Sci. U.S.A..

[18] Iwahara J., Clore G.M. (2006). Direct observation of enhanced translocation of a homeodomain between DNA cognate sites by NMR exchange spectroscopy. J. Am. Chem. Soc..

[19] Iwahara J., Clore G.M. (2006). Detecting transient intermediates in macromolecular binding by paramagnetic NMR. Nature.

[20] Givaty O., Levy Y. (2009). Protein sliding along DNA: dynamics and structural characterization. J. Mol. Biol..

[21] Proctor A.J., Stevens C.A., Cho S.S. (2013). GPU-Optimized Hybrid Neighbor / Cell List Algorithm for Coarse-Grained MD Simulations of Protein and RNA Folding and Assembly Categories and Subject Descriptors.

[22] Lipscomb T.J., Cho S.S. (2012). Parallel Verlet Neighbor List Algorithm for GPU-Optimized MD Simulations Categories and Subject Descriptors.

[23] Vuzman D., Polonsky M., Levy Y. (2010). Facilitated DNA search by multidomain transcription factors: cross talk via a flexible linker. Biophys. J..

[24] Vuzman D., Levy Y. (2010). DNA search efficiency is modulated by charge composition and distribution in the intrinsically disordered tail. Proc. Natl. Acad. Sci. U.S.A..

[25] Tóth-Petróczy A., Simon I., Fuxreiter M., Levy Y. (2009). Disordered tails of homeodomains facilitate DNA recognition by providing a trade-off between folding and specific binding. J. Am. Chem. Soc..

[26] Marcovitz A., Levy Y. (2013). Obstacles may facilitate and direct DNA search by proteins. Biophys. J..

[27] Guardiani C., Cencini M., Cecconi F. (2014). Coarse-grained modeling of protein unspecifically bound to DNA. Phys. Biol..

[28] Brackley C.A., Cates M.E., Marenduzzo D. (2013). Intracellular facilitated diffusion: Searchers, crowders, and blockers. Phys. Rev. Lett..

[29] Marklund E.G., Mahmutovic A., Berg O.G., Hammar P., van der Spoel D., Fange D., Elf J. (2013). Transcription-factor binding and sliding on DNA studied using micro- and macroscopic models. Proc. Natl. Acad. Sci. U.S.A..

[30] Hu T., Grosberg A.Y., Shklovskii B.I. (2006). How proteins search for their specific sites on DNA: the role of DNA conformation. Biophys. J..

[31] Brackley C.A., Cates M.E., Marenduzzo D. (2013). Effect of DNA conformation on facilitated diffusion. Biochem. Soc. Trans..

[32] Prévost C., Takahashi M., Lavery R. (2009). Deforming DNA: from physics to biology. Chemphyschem A Eur. J. Chem. Phys. Phys. Chem..

[33] Fogg J.M., Randall G.L., Pettitt B.M., Sumners D.W.L., Harris S.A., Zechiedrich L. (2012). Bullied no more: when and how DNA shoves proteins around. Q. Rev. Biophys..

[34] Bouvier B., Zakrzewska K., Lavery R. (2011). Protein-DNA recognition triggered by a DNA conformational switch. Angew. Chemie Int. Ed..

[35] Liu Z., Deibler R.W., Chan H.S., Zechiedrich L. (2009). The why and how of DNA unlinking. Nucleic Acids Res..

[36] Rohs R., Jin X., West S.M., Joshi R., Honig B., Mann R.S. (2010). Origins of specificity in protein-DNA recognition. Annu. Rev. Biochem..

[37] Joshi R., Passner J.M., Rohs R., Jain R., Sosinsky A., Crickmore M.A., Jacob V., Aggarwal A.K., Honig B., Mann R.S. (2007). Functional specificity of a Hox protein mediated by the recognition of minor groove structure. Cell.

[38] Rohs R., West S.M., Sosinsky A., Liu P., Mann R.S., Honig B. (2009). The role of DNA shape in protein-DNA recognition. Nature.

[39] Zhou T., Shen N., Yang L., Abe N., Horton J., Mann R.S., Bussemaker H.J., Gordân R., Rohs R. (2015). Quantitative modeling of transcription factor binding specificities using DNA shape.

[40] Barozzi I., Simonatto M., Bonifacio S., Yang L., Rohs R., Ghisletti S., Natoli G. (2014). Coregulation of transcription factor binding and nucleosome occupancy through DNA features of mammalian enhancers. Mol. Cell.

[41] Zhou T., Yang L., Lu Y., Dror I., Dantas Machado A.C., Ghane T., Di Felice R., Rohs R. (2013). DNAshape: a method for the high-throughput prediction of DNA structural features on a genomic scale. Nucleic Acids Res..

[42] Rohs R., West S.M., Liu P., Honig B. (2009). Nuance in the double-helix and its role in protein-DNA recognition. Curr. Opin. Struct. Biol..

[43] Bhattacherjee A., Levy Y. (2014). Search by proteins for their DNA target site: 1. The effect of DNA conformation on protein sliding. Nucleic Acids Res..

[44] Bhattacherjee A., Levy Y. (2014). Search by proteins for their DNA target site: 2. The effect of DNA conformation on the dynamics of multidomain proteins. Nucleic Acids Res..

[45] Chen Z., Yang H., Pavletich N.P. (2008). Mechanism of homologous recombination from the RecA-ssDNA/dsDNA structures. Nature.

[46] Luger K., Mäder A.W., Richmond R.K., Sargent D.F., Richmond T.J. (1997). Crystal structure of the nucleosome core particle at 2.8 A resolution. Nature.

[47] Halford S.E., Marko J.F. (2004). How do site-specific DNA-binding proteins find their targets. Nucleic Acids Res..

[48] Wilhelm M., Mukherjee A., Bouvier B., Zakrzewska K., Hynes J.T., Lavery R. (2012). Multistep drug intercalation: Molecular dynamics and free energy studies of the binding of daunomycin to DNA. J. Am. Chem. Soc..

[49] Zimmerman S.B., Trach S.O. (1991). Estimation of macromolecule concentrations and excluded volume effects for the cytoplasm of Escherichia coli. J. Mol. Biol..

[50] Ellis R.J. (2001). Macromolecular crowding: Obvious but underappreciated. Trends Biochem. Sci..

[51] Ellis R.J., Minton A.P. (2003). Cell biology: join the crowd. Nature.

[52] Benedetti F., Japaridze A., Dorier J., Racko D., Kwapich R., Burnier Y., Dietler G., Stasiak A. (2015). Effects of physiological self-crowding of DNA on shape and biological properties of DNA molecules with various levels of supercoiling. Nucleic Acids Res..

[53] Elf J., Berg O.G., Li G.-W. (2009). Effects of macromolecular crowding and DNA looping on gene regulation kinetics. Nat. Phys..

[54] Flyvbjerg H., Keatch S.A., Dryden D.T.F. (2006). Strong physical constraints on sequence-specific target location by proteins on DNA molecules. Nucleic Acids Res..

[55] Zhou H.-X. (2005). A model for the mediation of processivity of DNA-targeting proteins by nonspecific binding: dependence on DNA length and presence of obstacles. Biophys. J..

[56] Akabayov B., Richardson C.C. (2012). Impact of Macromolecular Crowding on DNA Replication. Biophys. J..

[57] Cravens S.L., Schonhoft J.D., Rowland M.M., Rodriguez A.A., Anderson B.G., Stivers J.T. (2015). Molecular crowding enhances facilitated diffusion of two human DNA glycosylases. Nucleic Acids Res..

[58] Tabaka M., Kalwarczyk T., Hołyst R. (2014). Quantitative influence of macromolecular crowding on gene regulation kinetics. Nucleic Acids Res..

[59] Clementi C., Nymeyer H., Onuchic J.N. (2000). Topological and energetic factors: what determines the structural details of the transition state ensemble and “en-route” intermediates for protein folding? An investigation for small globular proteins. J. Mol. Biol..

[60] Onuchic J.N., Wolynes P.G. (2004). Theory of protein folding. Curr. Opin. Struct. Biol..

[61] Azia A., Levy Y. (2009). Nonnative Electrostatic Interactions Can Modulate Protein Folding: Molecular Dynamics with a Grain of Salt. J. Mol. Biol..

[62] Schlick T. (2000). Molecular Modeling and Simulation: An Interdisciplinary Guide.

[63] Li R., Ge H.W., Cho S.S. (2013). Sequence-dependent base-stacking stabilities guide tRNA folding energy landscapes. J. Phys. Chem. B.

[64] Hyeon C., Thirumalai D. (2005). Mechanical unfolding of RNA hairpins. Proc. Natl. Acad. Sci. U.S.A..

[65] Biyun S., Cho S.S., Thirumalai D. (2011). Folding of human telomerase RNA pseudoknot using ion-jump and temperature-quench simulations. J. Am. Chem. Soc..

[66] Pincus D.L., Cho S.S., Hyeon C., Thirumalai D. (2008). Minimal Models for Protein and RNA: From Folding to Function. Prog. Mol. Biol. Transl. Sci..

[67] Schlick T., Perisić O. (2009). Mesoscale simulations of two nucleosome-repeat length oligonucleosomes. Phys. Chem. Chem. Phys..

[68] Levy Y., Onuchic J.N., Wolynes P.G. (2007). Fly-casting in protein-DNA binding: frustration between protein folding and electrostatics facilitates target recognition. J. Am. Chem. Soc..

[69] Marcovitz A., Levy Y. (2009). Arc-repressor dimerization on DNA: folding rate enhancement by colocalization. Biophys. J..

[70] Marcovitz A., Levy Y. (2012). Sliding Dynamics Along DNA: A Molecular Perspective. Royal Soc. Chem..

[71] Sambriski E.J., Schwartz D.C., De Pablo J.J. (2009). A mesoscale model of DNA and its renaturation. Biophys. J..

[72] Terakawa T., Kenzaki H., Takada S. (2012). p53 searches on DNA by rotation-uncoupled sliding at C-terminal tails and restricted hopping of core domains. J. Am. Chem. Soc..

[73] Takagi F., Koga N., Takada S. (2003). How protein thermodynamics and folding mechanisms are altered by the chaperonin cage:Molecular Simulations. Proc. Natl. Acad. Sci. U.S.A..

[74] Wang W., Xu W., Levy Y., Trizac E., Wolynes P.G. (2009). Confinement effects on the kinetics and thermodynamics of protein dimerization. Proc. Natl. Acad. Sci. U.S.A..

[75] Zheng G., Lu X.-J., Olson W.K. (2009). Web 3DNA–a web server for the analysis, reconstruction, and visualization of three-dimensional nucleic-acid structures. Nucleic Acids Res..

[76] Marcovitz A., Levy Y. (2013). Weak Frustration Regulates Sliding and Binding Kinetics on Rugged Protein-DNA Landscapes.

[77] Stella S., Cascio D., Johnson R.C. (2010). The shape of the DNA minor groove directs binding by the DNA-bending protein Fis.

[78] Ando T., Skolnick J. (2014). Sliding of proteins non-specifically bound to DNA: Brownian dynamics studies with coarse-grained protein and DNA models. PLoS Comput. Biol..

[79] Slutsky M., Kardar M., Mirny L. (2004). Diffusion in correlated random potentials, with applications to DNA. Phys. Rev. E.

[80] Erickson H.P. (2009). Size and shape of protein molecules at the nanometer level determined by sedimentation, gel filtration, and electron microscopy. Biol. Proced. Online.

