# Supplementary material for: Searching target sites on DNA by proteins: Role of DNA dynamics under confinement
Source: Nucleic Acids Res. 2015 Sep 22;43(19):9176–86. doi: 10.1093/nar/gkv931 (PMC4627088; doi:10.1093/nar/gkv931)
Supplement: SUPPLEMENTARY DATA [file supp_43_19_9176__index.html]

Searching target sites on DNA by proteins: Role of DNA dynamics under confinement — Searching target sites on DNA by proteins: Role of DNA dynamics under confinement — SUPPLEMENTARY DATA 

# Searching target sites on DNA by proteins: Role of DNA dynamics under confinement

## SUPPLEMENTARY DATA

- SUPPLEMENTARY DATA
