# Supplementary material for: Searching target sites on DNA by proteins: Role of DNA dynamics under confinement
Source: Nucleic Acids Res. 2015 Sep 22;43(19):9176–86. doi: 10.1093/nar/gkv931 (PMC4627088; doi:10.1093/nar/gkv931)
Supplement: SUPPLEMENTARY DATA [file supp_gkv931_nar-01526-f-2015-File009.pdf]

Supplementary for
Searching target sites on DNA by proteins: Role of DNA dynamics under confinement

Anupam Mondal[1], and Arnab Bhattecherjee[1,*]

Supplementary text

Protein Model:

To represent the molecular nature of the DBPs, we performed coarse-Grained molecular dynamics simulation of Sap-1 where each residue corresponds to an amino acid, located at C_α position. The potential energy of protein molecule is obtained from:

$$E_{pot} = E_{bond} + E_{bend} + E_{torsion} + E_{LJ} + E_{ev} + E_{ele}$$

E_{bond} represents bonded energy and is given by,

$$E_{bond} = \sum_i k_b (r_i - r_i^0)^2$$

where k_b =100.0, r_i and r_i^0 are the distances between i-th and i+1-th C_α beads in intermediate and folded structures respectively.

E_{bend} is the potential energy function for variation of angles and is given by,

$$E_{bend} = \sum_i k_\theta (\theta_i - \theta_i^0)^2$$

where k_θ =20.0, θ_i and θ_i^0 are the angles among i-th, i+1-th, i+2-th C_α beads in intermediate and folded structure respectively.

$E_{torsion}$ is the potential energy function for torsional angle formed by every four atoms connected by chemical bonds and is given by,

$$E_{torsion} = \sum_i \{k_{\phi 1}[1-\cos 3(\phi_i - \phi_i^0)] + k_{\phi 2}[1-\cos(\phi_i - \phi_i^0)]\}$$

where $k_{\phi 1}$ =0.5, $k_{\phi 2}$ =1.0, ϕ_i and ϕ_i^0 are the torsional angle among i-th, i+1-th, i+2-th, i+3-th C_α beads in intermediate and folded structures respectively.

E_{LJ} is the Lennard-Jones potential energy function for stabilizing protein's folded structure and native contact interactions and is given by,

$$E_{LJ} = \sum_{i<j-3}^{native} \varepsilon_{ij} \left[5\left(\frac{\sigma_{ij}}{r_{ij}}\right)^{12} - 6\left(\frac{\sigma_{ij}}{r_{ij}}\right)^{10} \right]$$

where ε_{ij} =4.0, r_{ij} represents the distance between native pairs at a given time and σ_{ij} =4.0 is the radius of interacting beads.

E_{ev} denotes the excluded volume effect between all non-bonded and non-native pairs and is given by,

$$E_{ev} = \sum_{i<j-3}^{non-native} \varepsilon_{ev} \left(\frac{\sigma_{ij}}{r_{ij}}\right)^{12}$$

where ε_{ev} =4.0, r_{ij} is the distance between i-th and j-th beads and $\sigma_{ij} = \sigma_i + \sigma_j$ is the interaction specific length scale with σ_i and σ_j are the respective radii of the interacting beads

E_{ele} is the potential energy function for electrostatic interaction between a pair of atoms and is represented by Debye-Huckel potential,

$$E_{ele} = \sum_{i<j} \frac{q_i q_j}{4\pi \varepsilon_0 \varepsilon_k r_{ij}} e^{-r_{ij}/\lambda_D}$$

Where $\lambda_D = \left(\frac{\varepsilon_0 \varepsilon_k k_B T}{2 N_A e^2 I}\right)^{0.5}$ is Debye length correspond to charge screening between the separation r_{ij}, q_i is the charge on i-th atom, ε_0 is permittivity of free space, ε_k =78.0 is the dielectric constant, $k_{\mathbf{B}}$ is the Boltzmann constant, T is temperature, N_A is Avogadro number, e is the elementary charge and I is the ionic strength of the solution.

DNA Model:

To describe DNA force field, we adopted 3SPN.1 model developed by Pablo et. al. In the 3SPN.1 model, the double stranded DNA molecules are composed of sugar, phosphate group and nitrogenous base (where nitrogenous base are either A, T, G, C) that are located at the geometric center of the associated group. In 3SPN.1 model, we incorporated the potential energy function as follows:

$$E_{pot}^{DNA} = E_{bond}^{DNA} + E_{bend}^{DNA} + E_{torsion}^{DNA} + E_{stack}^{DNA} + E_{base}^{DNA} + E_{solv}^{DNA} + E_{ev} + E_{ele} + E_{sp}$$

E_{bond}^{DNA} is the potential energy function for covalent bonding interactions and is given by,

$$E_{bond}^{DNA} = \sum_i \left[k_1 \left(r_i - r_i^0 \right)^2 + k_2 \left(r_i - r_i^0 \right)^4 \right]$$

where k_1 =0.1839, k_2 =183.9 and r_i, r_i^0 are respectively the instantaneous and equilibrium bond length for the i-th bond.

E_{bend}^{DNA} is the potential energy function for molecular bending and is given by,

$$E_{bend}^{DNA} = \sum_i k_\theta \left(\theta_i - \theta_i^0 \right)^2$$

where k_θ =128.73 and θ_i θ_i^0 are respectively the instantaneous and equilibrium bond angles for the i-th bond angle.

$E_{torsion}^{DNA}$ is the potential energy function for torsional angle formed by every four atoms connected by chemical bonds and is given by,

$$E_{torsion}^{DNA} = \sum_i k_\phi \left[1 - \cos\left(\phi_i - \phi_i^0 \right) \right]$$

where k_ϕ =5.1492 and ϕ_i, ϕ_i^0 are respectively the instantaneous and equilibrium dihedral angles for the i-th dihedral angle.

E_{stack}^{DNA} is the potential energy function for intra-stand Base-stacking and is given by,

$$E_{stack}^{DNA} = \sum_{i<j} 4\varepsilon \left[\left(\frac{\sigma_{ij}}{r_{ij}} \right)^{12} - \left(\frac{\sigma_{ij}}{r_{ij}} \right)^{6} \right]$$

where ε =1.0, r_{ij} is the distance between i-th and j-th beads, σ_{ij} is the interaction-specific length scale. The summation is taken over all native contact pairs that are in the intra-stand DNA and for each pair of bead i and j satisfying |i-j|>2, a native contact is defined as when r_{ij} < 9 Å in the folded structure.

E_{base}^{DNA} is the potential energy function for base-pairing and is given by,

$$E_{base}^{DNA} = \sum_{i<j} 4\varepsilon_{bi} \left[5\left(\frac{\sigma_{bi}}{r_{ij}} \right)^{12} - 6\left(\frac{\sigma_{bi}}{r_{ij}} \right)^{10} \right]$$

where the summation is taken over all complementary base pairs that do not participate in E_{stack}^{DNA} and for each i-th base pair, r_{ij} represents the separation between intra-stand and inter-stand sites i and j in which the characteristic energies are $\varepsilon_{bi} = \varepsilon_{AT}$ or ε_{GC} and characteristic lengths are $\sigma_{bi} = \sigma_{AT}$ or σ_{GC} satisfying $\varepsilon_{\alpha\beta}$ = $\varepsilon_{\beta\alpha}$ and $\sigma_{\alpha\beta}$ = $\sigma_{\beta\alpha}$. We used ε_{bi}=0.3678, σ_{bi}=2.9002 for AT pairs and ε_{bi}=0.4656, σ_{bi}=2.8694 for GC pairs.

E_{solv}^{DNA} is the potential energy function for solvent-induced interaction over the inter-stand sugar pairs and is given by,

$$E_{solv}^{DNA} = \sum_{i<j} \varepsilon_s \left[1 - e^{-\alpha\left(r_{ij} - r_s\right)}\right]^2 - \varepsilon_s$$

where α=5.333, r_s =13.38 are constants and ε_s depends on the salt concentration.

The potential energy function for protein-DNA steric clashes (E_{ev}) and electrostatics interactions (E_{ele}) are of the same form as described in “Protein Model”.

The specific protein-DNA contact formation is ensured by a soft attractive Lennard-Jones potential with ε_{ij} =0.1.

Supplementary Figures:

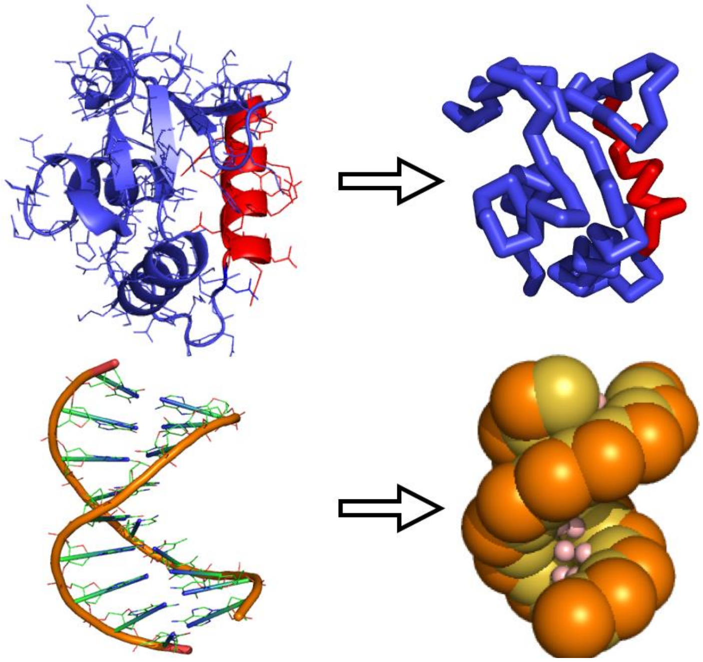

Figure S1: The overall structure of the Sap-1 and DNA molecules in fully atomistic model (top and bottom, left) and coarse-grained (top and bottom, right) representations. The recognition region for Sap-1 are labelled with red color that corresponds to 53-68 residue region in Sap-1. Each nucleotide in DNA is presented through three beads namely; negatively charged phosphate beads (orange color), yellow sugar beads and the small pink colored base beads.

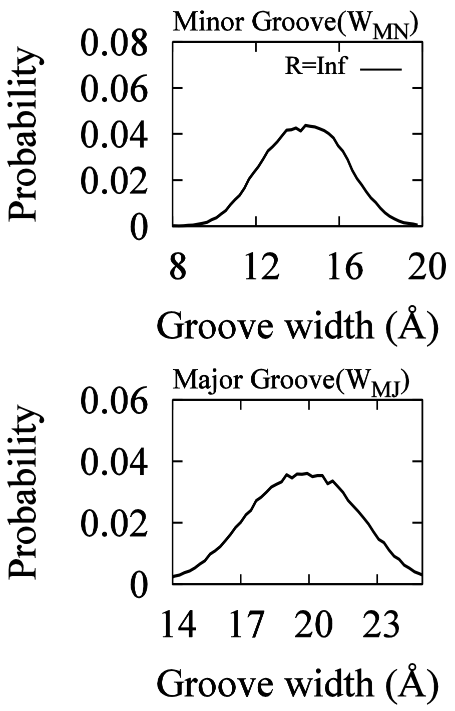

Figure S2: The representative histogram provides the fluctuation in the minor (top) and major (bottom) groove widths for without any confinement effect.

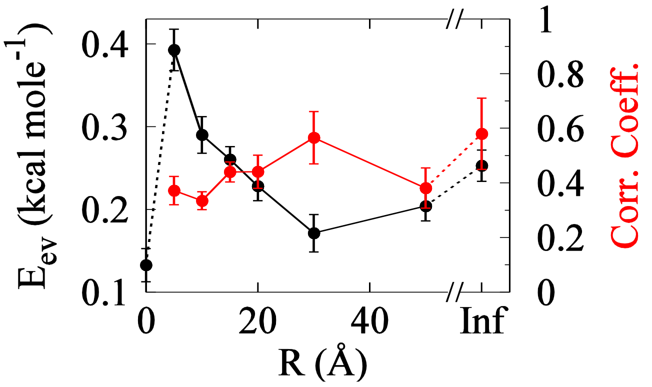

Figure S3: Variation of steric clashes (E_{ev}, denoted black lines) and correlation in dynamics of interacting molecules (red line) as function of intracellular space R at Cs=100mM. Depending on the degree of DNA confinement (indicated by R), these two parameters jointly determine the inter-communication between protein and DNA molecules.

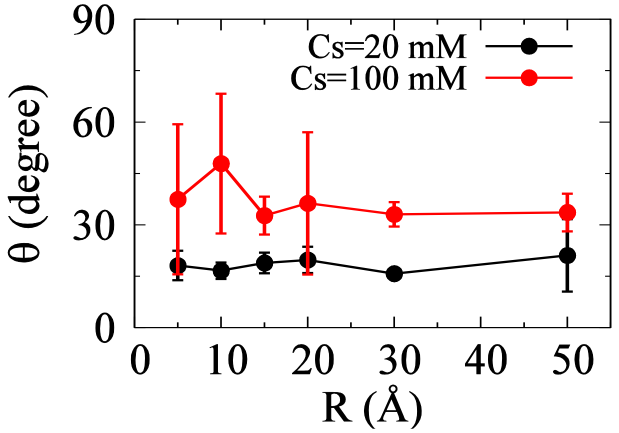

Figure S4: Change in the orientation angle (ψ) adopted by the DBPs around the DNA contour as a function of intracellular space R at salt concentration 20mM (black) and 100mM (red). At 100mM, corresponding electrostatic interactions are weak and inadequate to direct the recognition region of Sap-1 to orient toward DNA molecule. This is reflected from associated larger error bars in ψ that signifies degree of randomness in the orientation angle.

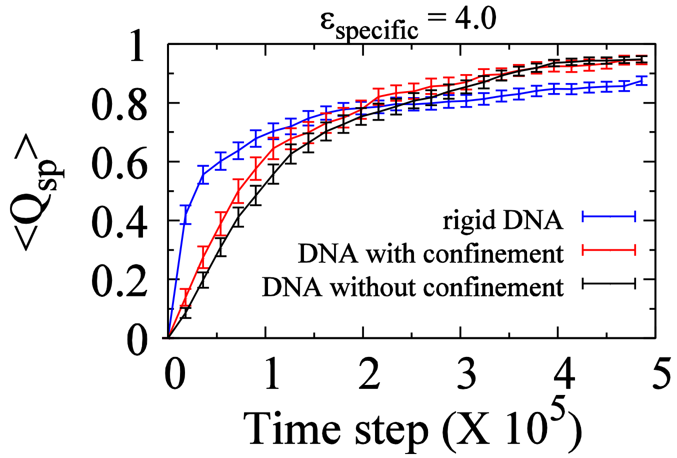

Fig S5: Kinetics of transition from non-specific to specific protein-DNA complex formation under strong binding affinity ($\varepsilon_{specific}$ =4.0). The fraction of number of specific contacts (Q_{sp}) as a function of time corresponding to rigid DNA (blue line), dynamic DNA under strong confinement (R=15 Å, red line) and dynamic DNA without confinement (black line) are calculated for without any nonspecific interaction between Sap-1 and DNA bases. Strong binding affinity at the target DNA site ensures formation of stable protein-DNA complex unlike to the situation presented in manuscript with $\varepsilon_{specific}$ =1.0.
